# Psychological distress and compliance with sanitary measures during the Covid-19 pandemic

**DOI:** 10.1371/journal.pone.0317272

**Published:** 2025-07-31

**Authors:** Irwin Hecker, Solène Wallez, Honor Scarlett, José Luis Ayuso-Mateos, Richard Bryant, Giulia Caggiu, Claudia Conflitti, Katalin Gémes, Josep Maria Haro, Vincent Lorant, Roberto Mediavilla, Ellenor Mittendorfer-Rutz, Anna Monistrol-Mula, Matteo Monzio Compagnoni, Papoula Petri-Romão, Irene Pinucci, Marit Sijbrandij, Jutta Stoffers-Winterling, Henrik Walter, Murielle Mary-Krause, Maria Melchior

**Affiliations:** 1 Sorbonne Université, INSERM, Institut Pierre Louis d’Épidémiologie et de Santé Publique, IPLESP, Équipe de Recherche en Épidémiologie Sociale, Paris, France; 2 Department of Psychiatry, Universidad Autónoma de Madrid, Madrid, Spain; 3 Department of Psychiatry, Hospital Universitario La Princesa - Instituto de Investigación Sanitaria, Madrid, Spain; 4 Centro de Investigación Biomédica en Red de Salud Mental (CIBERSAM), Instituto de Salud Carlos III, Madrid, Spain; 5 University of New South Wales, Australia; 6 Department of Statistics and Quantitative Methods, University of Milano-Bicocca, Milan, Italy; 7 National Centre for Healthcare Research and Pharmacoepidemiology, Department of Statistics and Quantitative Methods, University of Milano-Bicocca, Milan, Italy; 8 Department of Mental Health and Addiction Services, Lecco, Italy; 9 Department of Clinical Neuroscience, Division of Insurance Medicine, Karolinska Institutet, Stockholm, Sweden; 10 Parc Sanitari Sant Joan de Deu, Sant Joan de Déu Research Institute, Barcelona, Spain; 11 Department of Medicine, Universitat de Barcelona, Barcelona, Spain; 12 Institute of Health and Society, UCLouvain, Belgium; 13 Group of Epidemiology of Mental Disorders and Ageing, Sant Joan de Déu Research Institute, Barcelona, Spain; 14 Leibniz Institute for Resilience Research (LIR), Mainz, Germany; 15 Department of Human Neurosciences, Sapienza University of Rome, Rome, Italy; 16 Department of Clinical, Neuro and Developmental Psychology, WHO Collaborating Centre for Research and Dissemination of Psychological Interventions, Amsterdam Public Health research institute, Vrije Universiteit Amsterdam, Netherlands; 17 University Medical Center of the Johannes Gutenberg University Mainz, Mainz, Germany; 18 Department of Psychiatry and Psychotherapy, Charité-Universitätsmedizin Berlin, corporate member of Freie Universität Berlin and Humboldt-Universität zu Berlin, Germany; Aix-Marseille Universite, FRANCE

## Abstract

**Background:**

This study aims to understand the relationship between the experience of psychological distress and compliance with COVID-19 sanitary measures. We testeed whether this relationship was modified by individuals’ gender and socioeconomic status (i.e., educational level and employment).

**Methods:**

Data from four European cohort studies (n = 13,635), were analysed using an Individual Participant Data (IPD) meta-analytic approach. Mixed effect models were employed to examine associations between mental health difficulties and compliance with sanitary measures, as well as effect modification by socioeconomic status. Statistical models were stratified by gender.

**Results:**

We found a statistically significant association between mental health difficulties and increased compliance with sanitary measures in women, while amongst men the statistically significant association observed was opposite. Moreover, there was a statistically significant interaction between participants’ educational level and mental health difficulties amongst men only, indicating especially low compliance levels with COVID-19 sanitary measures amongst individuals with only primary schooling and who reported psychological distress.

**Conclusion:**

The association between psychological distress and compliance with sanitary measures is complex–positive in women, negative in men. Men experiencing mental health difficulties, especially those with lower educational attainment, exhibit low levels of compliance with sanitary measures. These results suggest that psychological distress and its possible consequences should be considered when designing measures addressing infectious disease spread.

## Introduction

Echoing patterns seen during prior epidemics [1], the COVID-19 pandemic showed widespread adverse impacts on global mental health [2–6]. The implementation of sanitary measures played a crucial role in mitigating the pandemic and preserving population [7]. Once sanitary measures are introduced, compliance is critical and determines their effectiveness and epidemiological impact [8].

Psychological distress, defined as experience of symptoms of depression, anxiety, insomnia, and multiple psychosocial complaints, which do not necessarily correspond to established diagnoses of psychiatric disorders but nevertheless lead to suffering and reduced quality of life, could be related to various aspects of individuals’ health behaviours, including compliance with sanitary measures at times of a health crisis. However the nature of the relationship between psychological distress and compliance with sanitary measures during a pandemic such as COVID-19 is complex from a theoretical perspective and past studies have yielded inconsistent findings. Symptoms of anxiety could lead individuals to be especially cautious and implement all sanitary measures with diligence, as has been shown among persons with mental health problems prior to the occurrence of the COVID-19 pandemic (for instance symptoms of depression), who were especially likely to implement social distancing, even as the pandemic subsided [9]. On the other hand, persons experiencing psychological distress might face socioeconomic difficulties, a factor associated with a reduced level of compliance with sanitary measures [10–12], which was observed during the COVID-19 pandemic [13,14].

Additionally, the COVID-19 pandemic worsened pre-existing gender inequalities. Women were more likely to experience psychological distress, including symptoms of anxiety and depression [15], before the pandemic, and the additional family charges experienced as a consequence of the health crisis (e.g., disproportionate caregiving burden during consecutive lockdowns, school closures) further amplified these pre-existing gender inequalities [16,17]. Employed women were at particularly high risk of having mental health difficulties given that they often carried the burden of domestic responsibilities, such as caring for children and the elderly, as well as changes in work-life balance [16,17]. On the other hand, women aged 35–39 years experienced higher levels of pandemic-induced unemployment, with an especially high risk in low educated women with young children [18].

Sanitary measures introduced to limit COVID-19 spread (e.g., lockdowns and business closures, remote work mandates, social distancing regulations, travel or healthcare access restrictions, school and university closures, and border closures) [19] had uneven economic and social impacts [20]. Whilst these measures were proven to mitigate COVID-19 outcomes, they exacerbated socioeconomic inequalities [21,22] due to heterogeneity in individuals’ ability to work remotely, or in the probability of COVID-19 infection within the household [23]. Individuals with no formal education and those with only primary education were less aware of available mental health care services [24]. Being employed [25] and having a low educational level [25–28] were also associated with non-compliance with sanitary measures–it may be because of other priorities such as securing means of subsistence [25], or the necessity to accept unsafe work conditions or even unstable income sources [25,29–31]. Regarding gender differences, behaviours also differ. More specifically women tend to be more compliant with precautionary measures (e.g., reduced mobility or wearing masks) than men [32].

We anticipated that compliance with sanitary measures during the COVID-19 pandemic would prove challenging for certain population groups. Specifically, we hypothesised that the association between compliance with sanitary measures and psychological distress would differ across genders and that educational level and employment status would further play a role in shaping these behaviours.

## Methods

### Study design and population

To investigate the relationship between the experience of psychological distress and compliance with sanitary measures, we combined data from multiple datasets collected during the COVID-19 pandemic into an Individual Participant Data (IPD). We employed IPD meta-analysis [33] utilising datasets exclusively sourced from partners within the RESPOND project. We carefully curated target variables and harmonised participating datasets to render the data comparable. Rigorous quality checks were conducted to ensure the reliability and integrity of all included data. This approach enhances the depth and reliability of our findings, enabling robust conclusions to be drawn from the combined datasets contributed by RESPOND project partners.

Data from four observational cohort studies were included: (i) the TEMPO (Trajectoires ÉpidéMiologiques en Population) [34], (ii) MINDCOVID [35], (iii) COVID and I [36], and (iv) the COVID-19 Mental Health Survey (COMET) [37].

The TEMPO cohort began in 2009 in France with the aim of better understanding mental health patterns and addictive behaviours. Starting from 2020, TEMPO participants were contacted to collect data regarding their health status during the COVID-19 pandemic. Nine waves of data were collected using self-administered questionnaires from March 24, 2020 (one week after the beginning of the first lockdown) to the end of July 2021. All COVID-19 study data waves were included in this study.

The MINDCOVID project is a survey of general population adults in Spain. The target population consisted of non-institutionalised Spanish adults (i.e., aged 18 years or older) without Spanish language barriers. Professional interviewers carried out computer-assisted telephone interviews (1–30 June 2020) in a sample drawn using dual-frame random digit dialling. Only the first and second waves of data collection were included in the present study, as compliance with sanitary measures was reported in these two study waves only.

COVID and I is an online survey conducted across Belgium through social media and national news outlets at the beginning of the first wave of the COVID-19 pandemic in 2020. The survey was launched on March 20^th^ 2020, two days after the beginning of lockdown. The survey was aimed at the general population and was available in English, French, and Dutch. Only the fourth wave of data collection in 2021 was included in this study as questions related to compliance with sanitary measures were asked at that wave only.

The COMET study is an international, online longitudinal survey aimed at evaluating the course of mental health symptoms during the COVID-19 pandemic, as well as identifying individuals at greater or reduced risk of mental illness. It includes participants from 14 countries (The Netherlands, Italy, Switzerland, Turkey, Spain, Germany, France, the United Kingdom, Sweden, South Africa, Indonesia, China, Australia and the United States). Participants were recruited starting in May 2020 through a snowball sampling strategy using university mailing lists and different social media platforms. Only the fourth and fifth waves of data collection in 2021 were included in the present study as questions related to compliance with sanitary measures were asked in these study waves only.

Data collection procedures are detailed in S1 Fig and population selection in S2 Fig. Other RESPOND databases (EDAD CON SALUD, HEROES, LORA, MARP, DYNACORE-L) were not included because they did not include information regarding compliance with sanitary measures (EDAD CON SALUD, HEROES, LORA, MARP) or mental health (DYNACORE-L).

### Ethics

All participating studies were approved by the appropriate ethics committee and were performed in accordance with the ethical standards laid down in the 1964 Declaration of Helsinki and its later amendments.

The TEMPO cohort received approval of bodies supervising ethical data collection in France, the Advisory Committee on the Treatment of Information for Health Research (Comité consultatif sur le traitement de l’information en matière de recherche dans le domaine de la santé, CCTIRS) and the French regulatory data protection authority (Commission Nationale de l’Informatique et des Libertés, CNIL, n◦ 908163).

The MINDCOVID study protocol was approved by the IRB Parc de Salut Mar (2020/9203/I) and by the corresponding IRBs of all the participating centres. The study is registered at ClinicalTrials.gov (https://clinicaltrials.gov/ct2/show/NCT04556565).

Covid and I’s ethical review and approval were not required as it is a population-based, online survey with no collection of personal data. Participants were provided with study information and online informed consent was obtained from all study participants.

The COMET study was approved by the ethical review board of the Faculty of Behavioral and Movement Sciences of the Vrije Universiteit Amsterdam (VCWE-2020–077). Personal data are protected according to EU and national laws.

All data included in the present analyses were fully anonymized.

### Measures

#### Outcome.

Compliance with sanitary measures was ascertained using self-reported items (handwashing, social distancing, physical contact, wearing a mask, lockdown, working from home, limiting small and/or large gatherings, curfew, hosting people at home, quarantine, and taking extra precautions with at risk people). These items were harmonised and summed (S1 Table); z-scores were calculated in each study sample.

### Exposure

Psychological distress was measured using the following psychological scales: COMET: Patient Health Questionnaire Anxiety and Depression Scale (PHQ-ADS) [38]; COVID and I: General Health Questionnaire-12 (GHQ-12) [39]; MINDCOVID: Patient Health Questionnaire-8 and the Anxiety and Depression Scale (PHQ8-ADS) [40]; TEMPO: Adult Self-Report (ASR)) [41]; these were harmonised using a standard procedure: first, for each scale all items were summed (S2 Table); next corresponding z-scores were calculated. In this way, individual values of each single scale across the four studies were transformed into a measure of the same order of magnitude (numeric, with specific minimum and maximum), making them comparable across samples.

### Covariates

Participants’ sociodemographic characteristics were collected across the four cohort studies included, coded consistently using the same names, values, and formats and included: gender (“Female”; “Male”), age, education (“Tertiary”; “Primary”; “Secondary”), employment status (“In employment”; “Unemployed”), and number of children (“No children”; “One”; “Two or three”; “Four or more”).

All variables accounted for in this study had previously been reported as factors associated with compliance with sanitary measures: female gender [28,42–46], age [26,43,44,46–50], educational level [25–28], employment status [51] and the number of children [52].

A “stringency” variable was also included, based on the Stringency Index [53], which incorporates nine metrics: school, workplace, or public transport closures, the cancellation of public events, restrictions on public gatherings, restrictions on internal movements, stay-at-home requirements, public information campaigns, and international travel controls. The score corresponds to the mean score of these nine metrics, with each metric ranging from 0 to 100. A higher number on the Stringency Index represents a stricter response to the COVID-19 pandemic, with 100 indicating the strictest possible measures.

### Statistical analysis

To test associations between participants’ psychological distress and compliance with the sanitary measures implemented during the COVID-19 pandemic, individual participant data from relevant studies was merged and analysed. Due to the longitudinal nature of the data, mixed effect models were used to calculate adjusted odds ratios (aOR), and the corresponding 95% confidence intervals (CI). Multivariate mixed effect models were adjusted for the above-listed covariates (please see the “Covariates” subsection). Analyses were conducted individually in each database, as well as in the merged dataset. We did not conduct country-specific analyses of the COMET data, as sample sizes are too small [54]. Data were stratified by gender to consider gender-specific patterns related to the outcome, exposure, and socioeconomic status. Additionally, interactions between socioeconomic status and exposure were explored. P-values of less than 0.05 were considered to be statistically significant. Collinearity of model variables was explored and measured using Variance Inflation Factor (VIF) derivatives, namely generalised VIF (GVIF) and GVIF^(1/(2*Df)) [55–58]. After removing incomplete cases in terms of the outcome and exposure, we included 19,143 longitudinal observations (from 13,635 participants). Incomplete data on covariates, with an average of 5% missing data, were imputed using Multiple Imputations by Chained Equations (MICE) with Fully Conditional Specification (FCS), based on five multiple imputations [59,60]. All statistical analyses were performed using R version 4.2.3 and Rstudio version 2023.6.1.524 [61].

## Results

Participants of the four cohorts studied showed many similarities, including a higher proportion of women (68.9%), and higher rates of both tertiary education (83.7%) and employment (83.5%). Participants’ mean age in each cohort ranged from 40 to 46 years. Among TEMPO participants, there was a high proportion of individuals with two or three children (57.9%), while most participants in the COMET, COVID and I and MINDCOVID cohorts, had no children (respectively 56.6%, 40.9%, 54.8%). The mean Stringency Index score ranged from 55 for COMET to 81 for TEMPO (Table 1).

**Table 1:** Characteristics of COMET, COVID and I, Mind COVID, and TEMPO cohort studies, March 2020–August 2022, n = 13,635.

Characteristics	COMET (25.03%)^*1*^	COVID and I (30.97%)^*1*^	MINDCOVID (23.58%)^*1*^	TEMPO (20.42%)^*1*^	All datasets^*1*^
N	3835	5935	2974	891	13,635
Gender					
Female	79.6%	74.7%	53.1%	65.3%	68.9%
Male	20.4%	25.3%	46.9%	34.7%	31.1%
Age in years	42 (16)	44 (12)	46 (14)	40 (4)	43 (12)
Education					
Tertiary	75.0%	89.3%	80.4%	89.5%	83.7%
Primary	5.6%	0.3%	4.0%	0.0%	2.4%
Secondary	19.4%	10.3%	15.7%	10.5%	13.9%
Employment Status					
In employment	85.3%	85.6%	75.5%	87.6%	83.5%
Unemployed	14.7%	14.4%	24.5%	12.4%	16.5%
Number of Children					
No children	56.6%	40.9%	54.8%	24.0%	44.6%
One	14.2%	20.3%	18.2%	16.3%	17.5%
Two or three	26.5%	35.6%	26.1%	57.9%	35.6%
Four or more	2.7%	3.2%	0.8%	1.8%	2.2%
Stringency	55 (24)	62 (2)	60 (10)	81 (11)	64 (16)

^*1*^ *%; Mean (SD).*

**Fig 1:**
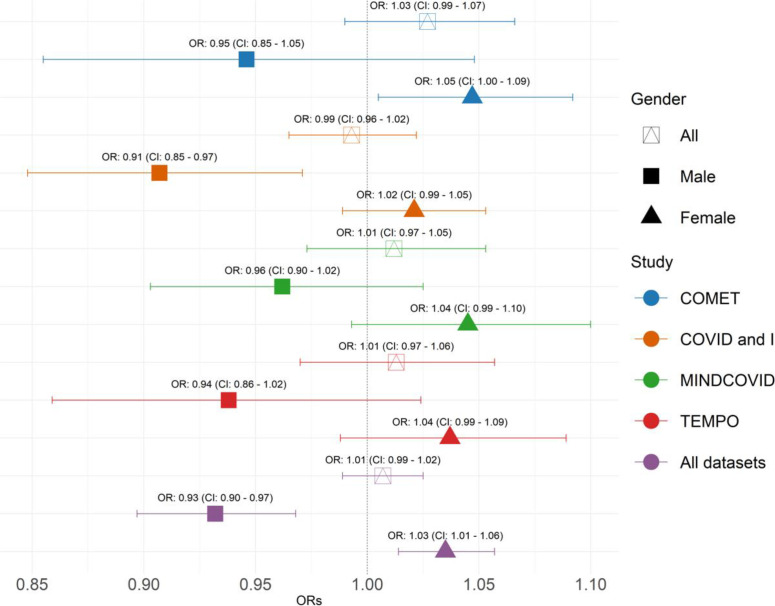
Association between psychological distress and compliance with sanitary measures in COMET, COVID and I, Mind COVID, TEMPO studies, and all populations, March 2020–August 2022, n = 13,635 (multivariate mixed models, adjusted odds-ratios (aOR), 95% confidence interval (CI)). All models adjusted for gender (except when stratified), age, education, employment status, number of children, and stringency. Both x and y axes show z scores.

When data were stratified by gender and adjusting for covariates, the association between psychological distress and compliance with sanitary measures was negative in men (aOR: 0.93, 95%CI: 0.90–0.97) and positive in women (aOR: 1.03, 95%CI: 1.01–1.06) (Fig 1).

**Fig 2:**
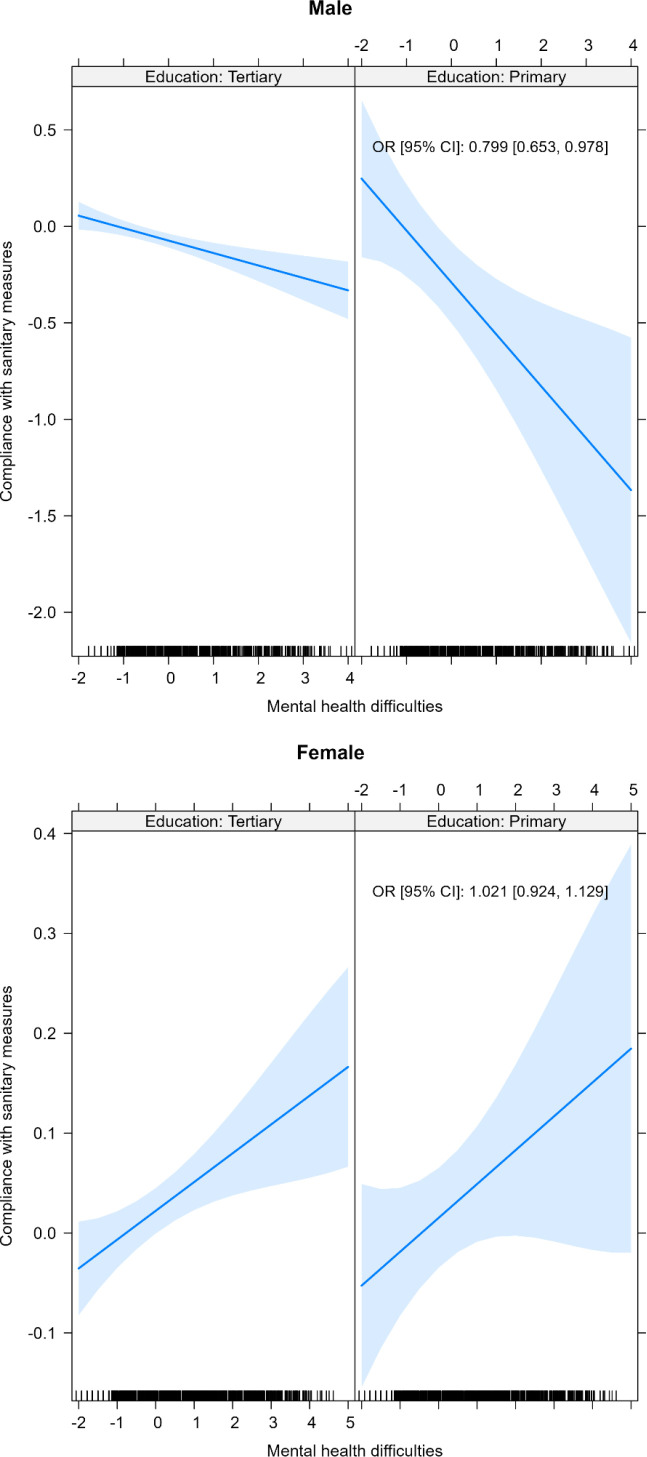
Interaction between participants’ educational level and psychological distress in relation to compliance with COVID-19 sanitary measures (COMET, COVID and I, Mind COVID, TEMPO studies), March 2020–August 2022, n = 13,635 (multivariate mixed models). All models adjusted for age, education, employment status, number of children, and stringency. Both x and y axes show z scores.

**Fig 3:**
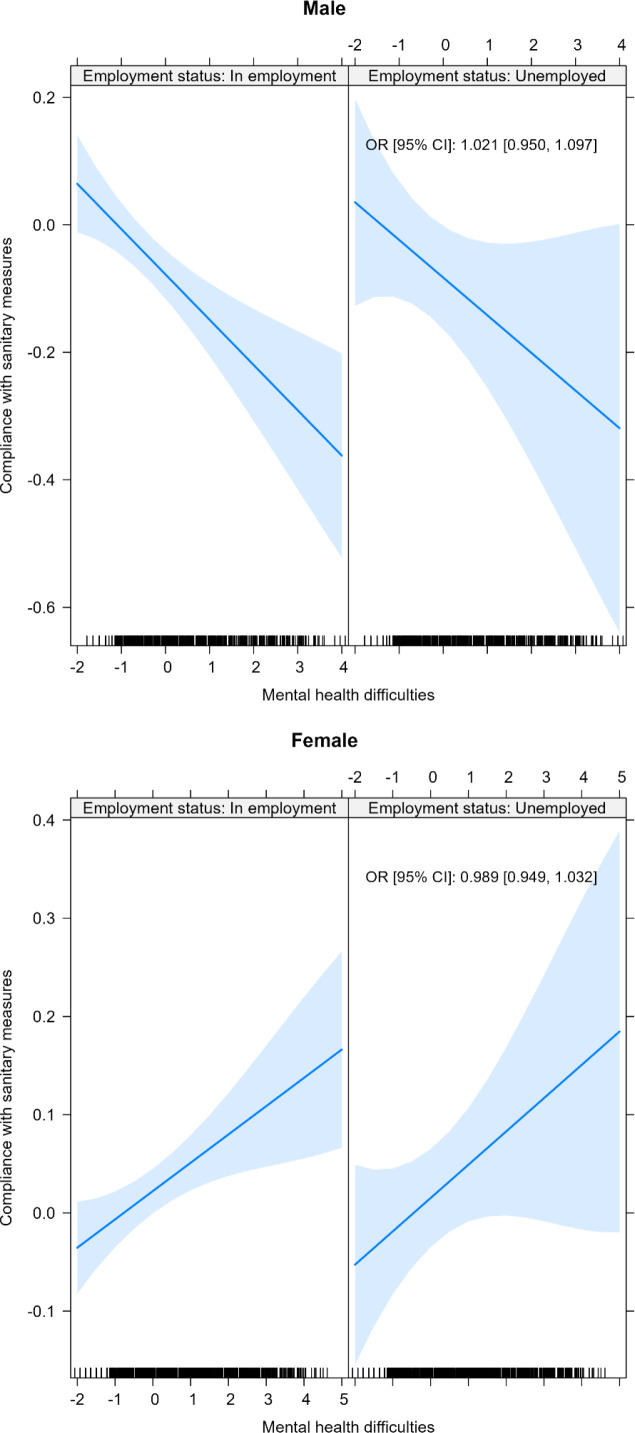
Interaction between participants’ employment status and psychological distress in relation to compliance with COVID-19 sanitary measures (COMET, COVID and I, Mind COVID, TEMPO studies), March 2020–August 2022, n = 13,635 (multivariate mixed models). All models adjusted for age, education, employment status, number of children, and stringency. Both x and y axes show z scores.

Additionally, we found a statistically significant interaction between psychological distress and primary educational level among men (aOR: 0.82, 95%CI: 0.68–0.99) (Fig 2), but not in other categories of education and employment status (Fig 3).

## Discussion

### Main findings of this study

Data collected among the 13,635 participants of the COMET, COVID and I, MINDCOVID and TEMPO cohorts from March 2020 to August 2022, revealed an association between psychological distress and compliance with sanitary measures during the COVID-19 period when stratified by gender. Women experiencing psychological distress showed higher compliance with sanitary measures, whereas the opposite relationship was observed amongst men. Additionally, an interaction between psychological distress and educational level among men was observed, such that compliance with sanitary measures was lowest in those with a low educational level.

### What is already known on this topic

The influence of both gender and socioeconomic characteristics on the association between psychological distress and compliance with sanitary measures is not yet well documented. Previous studies suggested that psychological distress has different effects on compliance with sanitary measures in men and women. Indeed, gender disparities in risk-taking behaviours [62] and health-related decision-making patterns during the COVID-19 pandemic have been documented [63]. Globally, men are more likely to engage in risky behaviours and less inclined to seek preventive medical care, or support for health issues [64–66]. During the pandemic, men perceived the consequences of COVID-19 to be less severe compared to women, despite objective evidence suggesting otherwise. Traditional masculinity norms appear to moderate this perception, which, in turn, negatively affects adherence to precautionary measures [67].

### What this study adds

Our study has a number of strengths worth highlighting. A significant strength of our study lies in the IPD meta-analytical approach, which enabled us to include four large cohort studies. IPD meta-analyses are recognized for providing a more comprehensive assessment of pooled data when compared to aggregate data analyses [68]. This methodology also allowed us to extract and analyse raw data from each individual study, including diverse spatial and temporal contexts throughout the COVID-19 pandemic, and thus enhance the precision and robustness of our findings by considering various contexts of data collection.

We observed an interaction between psychological distress and educational level in terms of compliance with COVID-19 sanitary measures was observed among men. This suggests an especially low level of compliance with sanitary measures in this group. It is known that bi-directional effects between academic achievement and social withdrawal exist in boys, increasing the risk of psychosocial maladjustment, depression, loneliness and anxiety [69]. It may also be that men and particularly those belonging to low educated groups face a number of challenges in terms of work and daily life, which make it less likely for them to invest in their health. This may partially explain the subsequent difficulty in complying with precautionary health measures in case of a pandemic.

Containment policies have resulted in a reduction of the impact of COVID-19 on population health [70,71]. Sanitary measures were proven to contain the spread of the virus [71,72], showing a linear, inverse relationship between the incidence of COVID-19 and degree of observed prevention measures [73]. During a health crisis, awareness of groups with low compliance to sanitary measures can be of help when intervening proactively, especially considering that shorter lockdown periods can be compensated for by high adherence to health-oriented interventions [8]. It is, then, important to educate the public about the negative consequences of the virus and the effectiveness of sanitary measures [74]. Regarding mental health, recommendations should aim to reduce mental health inequalities between vulnerable groups and the general population using measures targeted adapted to specific contexts [75].

As has previously been recommended, a gender-specific response to a new health risks emphasises the need for targeted public health messaging [32]. In a recent scoping review [63], it was revealed that individuals’ perceptions of COVID-19 health information and recommendations, as well as their decisions regarding health matters, are significantly influenced by their level of education and health literacy. This is particularly important since the effectiveness of containment measures in a case such as a pandemic as COVID-19 relies on widespread public understanding and support. Targeting low educated men with tailored mental health interventions would not only help tackle psychological distress related to the pandemic but also promote compliance with sanitary measures, thus reducing the risk of infection [76]. Mental health interventions for vulnerable groups are currently being tested within the context of the COVID-19 pandemic and its aftermath [77–80].

### Study limitations

We need to acknowledge some limitations to our study. First, as the data were self-reported, it may contain biases stemming from social desirability and memory recall issues [81]. Second, some key variables, such as participants’ income [27,44,47,50,82], presence of a chronic illness [83], or COVID-19-related worries [26] were excluded due to their heterogeneity across all included studies. Additionally, we were not able to measure participants’ risk perceptions, which were not systematically assessed in all participating cohorts. However, this selective inclusion was a deliberate choice aimed at maintaining methodological consistency across studies and ensuring rigorous statistical analyses. By focusing on variables shared across different cohorts, we enhanced the internal validity of our study, thereby providing a more reliable synthesis of the available evidence. Both outcome and exposure data were derived from distinct validated scales and questions within each survey, which were subsequently pooled post-collection using methods distinct from the standard approaches for the respective scales. Third, this set of data is not comprehensive as it was not identified and selected systematically; rather, it was included from datasets provided by partners of the RESPOND project. Nevertheless, this approach allowed a harmonised comparison of different datasets.

## Conclusion

Our study underscores the need for targeted interventions related to compliance with preventive measures among persons experiencing psychological distress, particularly during health crises like the COVID-19 pandemic. Our findings highlight the importance of tailoring messaging and strategies to address the unique challenges faced by different populations. Targeting specific groups with lower rates of compliance through tailored messaging is essential for effective management of health crises, such as the COVID-19 pandemic. Men experiencing psychological distress, especially those with lower educational attainment, show limited compliance with sanitary measures. This calls for targeted approaches towards men experiencing psychological distress, especially those with lower educational attainment. By promoting individuals’ well-being, taking into account their mental health, gender, and socioeconomic characteristics, we may better prepare for future health crises.

## Supporting information

S1 Fig Data collected in the COMET, COVID and I, Mind COVID and TEMPO studies, March 2020–August 2022, n = 13,635. (DOCX)

S2 Fig Flow chart of COMET, COVID and I, Mind COVID, and TEMPO samples, March 2020–August 2022, n = 13,635. (DOCX)

S1 Table Items measuring compliance with sanitary measures against COVID-19 available in COMET, COVID and I, Mind COVID and TEMPO studies, March 2020–August 2022, n = 13,635. (DOCX)

S2 Table Measures of mental health available in the COMET, COVID and I, Mind COVID and TEMPO studies, March 2020–August 2022, n = 13,635. (DOCX)
