# Supplementary material for: Psychological distress and compliance with sanitary measures during the Covid-19 pandemic
Source: PLoS One. 2025 Jul 31;20(7):e0317272. doi: 10.1371/journal.pone.0317272 (PMC12312964; doi:10.1371/journal.pone.0317272)

Supplementary Figure 1: Data collected in the COMET, COVID and I, Mind COVID and TEMPO studies, March 2020 - August 2022, n=13,635.


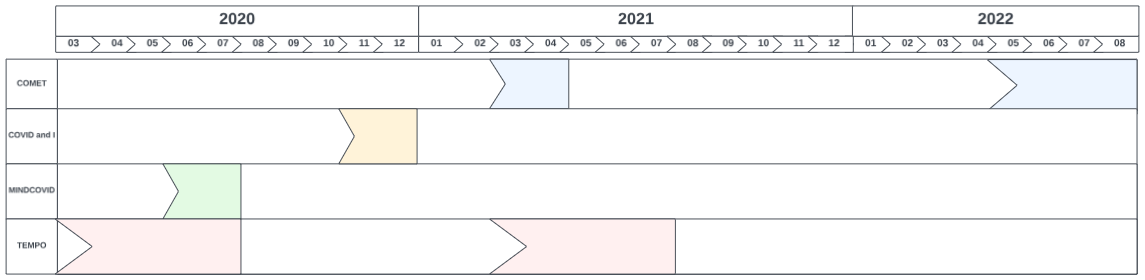

Supplement: S1 Fig — (DOCX) [file pone.0317272.s001.docx]
