# Supplementary material for: Psychological distress and compliance with sanitary measures during the Covid-19 pandemic
Source: PLoS One. 2025 Jul 31;20(7):e0317272. doi: 10.1371/journal.pone.0317272 (PMC12312964; doi:10.1371/journal.pone.0317272)
Supplement: S2 Fig — (DOCX) [file pone.0317272.s002.docx]

Supplementary Figure 2: Flow chart of COMET, COVID and I, Mind COVID, and TEMPO samples, March 2020 - August 2022, n=13,635.

*The study's longitudinal design allowed for the collection of data from participants at various intervals, providing, for some, multiple observations for analysis.*

COMET

TEMPO

MIND
COVID

COVID and I

8 084 individuals
40 420
observations

904 individuals
9 040
observations

3 500 individuals
10 500
observations

27 857 individuals
139 286
observations

Initial data import

7 938 individuals
15 876
observations

902
individuals
8 118
observations

3 500
individuals
7 000
observations

20 452
individuals
20 452
observations

Wave selection

3 835 individuals
4 783
observations

891
individuals
3 909
observations

2 974
individuals
4 516
observations

5 935 individuals
5 935
observations

13 635 individuals
19 143
observations

NA removal
