# Supplementary material for: Psychological distress and compliance with sanitary measures during the Covid-19 pandemic
Source: PLoS One. 2025 Jul 31;20(7):e0317272. doi: 10.1371/journal.pone.0317272 (PMC12312964; doi:10.1371/journal.pone.0317272)
Supplement: S1 Table — (DOCX) [file pone.0317272.s003.docx]

Supplementary Table 1: Items measuring compliance with sanitary measures against COVID-19 available in COMET, COVID and I, Mind COVID and TEMPO studies, March 2020 - August 2022, n=13,635.

| Study Name | Description | Question | Categories |
| --- | --- | --- | --- |
| COMET | Not being allowed to go outdoors, only with permission | How often did you adhere to the following regulations during the past week? | 1=Not at all  2=Most of the time  3=All of the time |
|  | Working from home |  |  |
|  | Restrictions related to small gatherings |  |  |
|  | Restrictions related to large gatherings |  |  |
|  | Wearing face masks in specific situations |  |  |
|  | Keeping a safe distance from people |  |  |
|  | Curfew |  |  |
|  | Being allowed to host only a limited number of people at home |  |  |
| COVID AND I | Maintain at least 1.5m distance from other people | To what extent have you complied with the following measures since their introduction? | 1=Strictly  2=Moderately  3=Scarcely  4=Not applicable |
|  | In case of illness: stay at home, do not go out shopping and/or receive visitors |  |  |
|  | Respect the “social bubble” (number of people allowed to meet with either at home or outside) |  |  |
|  | Take extra precautions with at-risk people |  |  |
|  | Cover your mouth and nose wherever it is compulsory |  |  |
|  | Cover your mouth and nose whenever at least 1.5 m distance from other people cannot be guaranteed |  |  |
| TEMPO Q1-Q7 | Increasing the frequency of handwashing (with soap or hand sanitiser) | What precautions do you take to avoid getting infected with Covid-19? Please tick all the boxes that apply to you. | 0 = Not ticked  1 = Ticked |
|  | Maintaining distance when passing by a stranger |  |  |
|  | Avoiding physical contact (including with family and friends) |  |  |
|  | Wearing latex gloves |  |  |
|  | Wearing a mask |  |  |
|  | Sneezing / coughing into your elbow |  |  |
|  | Avoiding public transport |  |  |
|  | Avoiding recreational and/or business trips |  |  |
| TEMPO Q8-Q9 | In public transport | In which situation do you respect shielding measures? Please tick every box that corresponds to your situation / Wearing a mask: |  |
|  | In the street |  |  |
|  | In shops |  |  |
|  | At work |  |  |
|  | When meeting friends |  |  |
|  | When meeting family |  |  |
|  | When meeting people at risk |  |  |
|  | In public transport | In which situation do you respect shielding measures? Please tick every box that corresponds to your situation / Handwashing: |  |
|  | In the street |  |  |
|  | In shops |  |  |
|  | At work |  |  |
|  | When meeting friends |  |  |
|  | When meeting family |  |  |
|  | When meeting people at risk |  |  |
|  | In public transport | In which situation do you respect shielding measures? Please tick every box that corresponds to your situation / Keeping a 1-metre distance: |  |
|  | In the street |  |  |
|  | In shops |  |  |
|  | At work |  |  |
|  | When meeting friends |  |  |
|  | When meeting family |  |  |
|  | When meeting people at risk |  |  |
|  | In public transport | In which situation do you respect shielding measures? Please tick every box that corresponds to your situation / No physical contact (hugging, kissing): |  |
|  | In the street |  |  |
|  | In shops |  |  |
|  | At work |  |  |
|  | When meeting friends |  |  |
|  | When meeting family |  |  |
|  | When meeting people at risk |  |  |
| MIND COVID | In the last 30 days, how do you rate your compliance with the restrictions imposed by the government? | | 1 = very low  2 = below average  3 = average  4 = above average  5 = very high |
