# Supplementary material for: Psychological distress and compliance with sanitary measures during the Covid-19 pandemic
Source: PLoS One. 2025 Jul 31;20(7):e0317272. doi: 10.1371/journal.pone.0317272 (PMC12312964; doi:10.1371/journal.pone.0317272)
Supplement: S2 Table — (DOCX) [file pone.0317272.s004.docx]

Supplementary Table 2: Measures of mental health available in the COMET, COVID and I, Mind COVID and TEMPO studies, March 2020 - August 2022, n=13,635.

| **Study Name** | **Scale** | **Question** | **Description** | **Categories** |
| --- | --- | --- | --- | --- |
| COMET | PHQ9-ADS  (PHQ-9 and GAD-7) | Over the past 2 weeks, how often have you been bothered by any of the following problems? | Little interest or pleasure in doing things? | 0 = Not at all  1 = Several days  2 = More than half the days  3 = Nearly every day |
|  |  |  | Feeling down, depressed, or hopeless? |  |
|  |  |  | Trouble falling or staying asleep, or sleeping too much? |  |
|  |  |  | Feeling tired or having little energy? |  |
|  |  |  | Poor appetite or overeating? |  |
|  |  |  | Feeling bad about yourself—or that you are a failure or have let yourself or your family down? |  |
|  |  |  | Trouble concentrating on things, such as reading the newspaper or watching television? |  |
|  |  |  | Moving or speaking so slowly that other people could have noticed? Or the opposite—being so fidgety or restless that you have been moving around a lot more than usual? |  |
|  |  |  | Thoughts that you would be better off dead or of hurting yourself in some way? |  |
|  |  |  | Feeling nervous, anxious, or on edge |  |
|  |  |  | Not being able to stop or control worrying |  |
|  |  |  | Worrying too much about different things |  |
|  |  |  | Trouble relaxing |  |
|  |  |  | Being so restless that it is hard to sit still |  |
|  |  |  | Becoming easily annoyed or irritable |  |
|  |  |  | Feeling afraid, as if something awful might happen |  |
| COVID AND I | GHQ-12 | Have you recently... | ...been able to concentrate on whatever you're doing? | The positive items were corrected from 0 (always) to 3 (never) and the negative ones were corrected from 0 (always) to 3 (never). |
|  |  |  | ...lost much sleep over worry? |  |
|  |  |  | ...felt that you are playing a useful part in things? |  |
|  |  |  | ...felt capable of making decisions about things? |  |
|  |  |  | ...felt constantly under strain? |  |
|  |  |  | ...felt you couldn't overcome your difficulties? |  |
|  |  |  | ...been able to enjoy your normal day-to-day activities? |  |
|  |  |  | ...been able to face up to your problems? |  |
|  |  |  | ...been feeling unhappy or depressed? |  |
|  |  |  | ...been losing confidence in yourself? |  |
|  |  |  | ...been thinking of yourself as a worthless person? |  |
|  |  |  | ...been feeling reasonably happy, all things considered? |  |
| TEMPO Q1 | ASR | Among the descriptions below, indicate how they have applied to you in the past 7 days by ticking one of the boxes. If some of the descriptions do not apply to you, answer by thinking of similar situations you have encountered. | I’m too forgetful | 0 = Not at all true  1 = Sometimes or a little true  2 = Very true or often true |
|  |  |  |  |  |
|  |  |  |  |  |
|  |  |  | I have trouble concentrating or paying attention for long |  |
|  |  |  |  |  |
|  |  |  |  |  |
|  |  |  | I can’t get my mind off of certain thoughts |  |
|  |  |  |  |  |
|  |  |  |  |  |
|  |  |  | I have trouble sitting still |  |
|  |  |  |  |  |
|  |  |  |  |  |
|  |  |  | I cry a lot |  |
|  |  |  |  |  |
|  |  |  |  |  |
|  |  |  | I deliberately try to hurt or kill myself |  |
|  |  |  |  |  |
|  |  |  |  |  |
|  |  |  | I worry about my future |  |
|  |  |  |  |  |
|  |  |  |  |  |
|  |  |  | I don't eat as well as I should |  |
|  |  |  |  |  |
|  |  |  |  |  |
|  |  |  | I'm afraid of certain animals (dogs, insects, etc.) or certain situations (elevators, heights, crowds, etc.) |  |
|  |  |  |  |  |
|  |  |  |  |  |
|  |  |  | I feel worthless or inferior |  |
|  |  |  |  |  |
|  |  |  |  |  |
|  |  |  | I accidentally get hurt a lot, accident-prone |  |
|  |  |  |  |  |
|  |  |  |  |  |
|  |  |  | I hear sounds or voices that other people think  aren’t there |  |
|  |  |  |  |  |
|  |  |  |  |  |
|  |  |  | I’m impulsive or act without thinking |  |
|  |  |  |  |  |
|  |  |  |  |  |
|  |  |  | I’m nervous or tense |  |
|  |  |  |  |  |
|  |  |  |  |  |
|  |  |  | Parts of my body twitch or make nervous  movements |  |
|  |  |  |  |  |
|  |  |  |  |  |
|  |  |  | I feel too guilty |  |
|  |  |  |  |  |
|  |  |  |  |  |
|  |  |  | I feel tired without good reason |  |
|  |  |  |  |  |
|  |  |  |  |  |
|  |  |  | My heart is pounding or racing with no known medical cause |  |
|  |  |  |  |  |
|  |  |  |  |  |
|  |  |  | I fail to finish things I should do |  |
|  |  |  |  |  |
|  |  |  |  |  |
|  |  |  | There is very little that I enjoy |  |
|  |  |  |  |  |
|  |  |  |  |  |
|  |  |  | My work/school performance is poor |  |
|  |  |  |  |  |
|  |  |  |  |  |
|  |  |  | I would rather be with older people than  with people of my own age |  |
|  |  |  |  |  |
|  |  |  |  |  |
|  |  |  | I repeat certain acts over and over |  |
|  |  |  |  |  |
|  |  |  |  |  |
|  |  |  | I hear sounds or voices that other people think  aren’t there |  |
|  |  |  |  |  |
|  |  |  |  |  |
|  |  |  | I worry about my family |  |
|  |  |  |  |  |
|  |  |  |  |  |
|  |  |  | I sleep more than most other people  during day and/or night |  |
|  |  |  |  |  |
|  |  |  |  |  |
|  |  |  | I have trouble making decisions |  |
|  |  |  |  |  |
|  |  |  |  |  |
|  |  |  | I do things that other people think are strange |  |
|  |  |  |  |  |
|  |  |  |  |  |
|  |  |  | I have thoughts that other people would think are strange |  |
|  |  |  |  |  |
|  |  |  |  |  |
|  |  |  | I rush into things without considering  the risks |  |
|  |  |  |  |  |
|  |  |  |  |  |
|  |  |  | I think about killing myself |  |
|  |  |  |  |  |
|  |  |  |  |  |
|  |  |  | I have trouble sleeping |  |
|  |  |  |  |  |
|  |  |  |  |  |
|  |  |  | I don't have much energy |  |
|  |  |  |  |  |
|  |  |  |  |  |
|  |  |  | I’m unhappy, sad, or depressed |  |
|  |  |  |  |  |
|  |  |  |  |  |
|  |  |  | People think I’m disorganised |  |
|  |  |  |  |  |
|  |  |  |  |  |
|  |  |  | I feel that I can’t succeed |  |
|  |  |  |  |  |
|  |  |  |  |  |
|  |  |  | I tend to lose things |  |
|  |  |  |  |  |
|  |  |  |  |  |
|  |  |  | I feel restless or fidgety |  |
|  |  |  |  |  |
|  |  |  |  |  |
|  |  |  | I’m too impatient |  |
|  |  |  |  |  |
|  |  |  |  |  |
|  |  |  | I’m not good at details |  |
|  |  |  |  |  |
|  |  |  |  |  |
|  |  |  | In this moment, I feel relaxed |  |
|  |  |  |  |  |
|  |  |  |  |  |
| TEMPO Q2-Q9 | ASR | Among the descriptions below, indicate how they have applied to you in the past 7 days by ticking one of the boxes. If some of the descriptions do not apply to you, answer by thinking of similar situations you have encountered. | I cry a lot | 0 = Not at all true  1 = Sometimes or a little true  2 = Very true or often true |
|  |  |  |  |  |
|  |  |  |  |  |
|  |  |  | I worry about my future |  |
|  |  |  |  |  |
|  |  |  |  |  |
|  |  |  | I don't eat as well as I should |  |
|  |  |  |  |  |
|  |  |  |  |  |
|  |  |  | I feel worthless or inferior |  |
|  |  |  |  |  |
|  |  |  |  |  |
|  |  |  | I’m impulsive or act without thinking |  |
|  |  |  |  |  |
|  |  |  |  |  |
|  |  |  | I am nervous or tense |  |
|  |  |  |  |  |
|  |  |  |  |  |
|  |  |  | Parts of my body twitch or make nervous  movements |  |
|  |  |  |  |  |
|  |  |  |  |  |
|  |  |  | I feel too guilty |  |
|  |  |  |  |  |
|  |  |  |  |  |
|  |  |  | I feel tired without good reason |  |
|  |  |  |  |  |
|  |  |  |  |  |
|  |  |  | My heart is pounding or racing with no known medical cause |  |
|  |  |  |  |  |
|  |  |  |  |  |
|  |  |  | I worry about my family |  |
|  |  |  |  |  |
|  |  |  |  |  |
|  |  |  | I think about killing myself |  |
|  |  |  |  |  |
|  |  |  |  |  |
|  |  |  | I have trouble sleeping |  |
|  |  |  |  |  |
|  |  |  |  |  |
|  |  |  | I'm unhappy, sad or depressed |  |
|  |  |  |  |  |
|  |  |  |  |  |
|  |  |  | I feel that I can't succeed |  |
|  |  |  |  |  |
|  |  |  |  |  |
|  |  |  | I feel restless or fidgety |  |
|  |  |  |  |  |
|  |  |  |  |  |
|  |  |  | I'm too impatient |  |
|  |  |  |  |  |
|  |  |  |  |  |
|  |  |  | At the moment I'm relaxed |  |
|  |  |  |  |  |
|  |  |  |  |  |
|  |  |  | I’m too forgetful |  |
|  |  |  |  |  |
|  |  |  |  |  |
|  |  |  | I'm afraid of certain animals (dogs, insects, etc.) |  |
|  |  |  |  |  |
|  |  |  |  |  |
|  |  |  | I'm afraid of certain situations (elevators, heights, crowds, etc.) |  |
|  |  |  |  |  |
|  |  |  |  |  |
|  |  |  | I’m afraid of doing wrong |  |
|  |  |  |  |  |
|  |  |  |  |  |
|  |  |  | I feel that no one loves me |  |
|  |  |  |  |  |
|  |  |  |  |  |
|  |  |  | I have seizures |  |
|  |  |  |  |  |
|  |  |  |  |  |
|  |  |  | I feel that others are out to get me |  |
|  |  |  |  |  |
|  |  |  |  |  |
|  |  |  | I lack self-confidence |  |
|  |  |  |  |  |
|  |  |  |  |  |
|  |  |  | I worry about my relations with the others |  |
|  |  |  |  |  |
|  |  |  |  |  |
|  |  |  | I worry about my social relations with the opposite sex |  |
|  |  |  |  |  |
|  |  |  |  |  |
| MIND COVID | PHQ8-ADS | Over the past 2 weeks, how often have you been bothered by any of the following problems? | Little interest or pleasure in doing things? | 0 = Not at all  1 = Several days  2 = More than half the days  3 = Nearly every day |
|  |  |  | Feeling down, depressed, or hopeless? |  |
|  |  |  | Trouble falling or staying asleep, or sleeping too much? |  |
|  |  |  | Feeling tired or having little energy? |  |
|  |  |  | Poor appetite or overeating? |  |
|  |  |  | Feeling bad about yourself—or that you are a failure or have let yourself or your family down? |  |
|  |  |  | Trouble concentrating on things, such as reading the newspaper or watching television? |  |
|  |  |  | Moving or speaking so slowly that other people could have noticed? Or the opposite—being so fidgety or restless that you have been moving around a lot more than usual? |  |
|  |  |  | Feeling nervous, anxious, or on edge |  |
|  |  |  | Not being able to stop or control worrying |  |
|  |  |  | Worrying too much about different things |  |
|  |  |  | Trouble relaxing |  |
|  |  |  | Being so restless that it is hard to sit still |  |
|  |  |  | Becoming easily annoyed or irritable |  |
|  |  |  | Feeling afraid, as if something awful might happen |  |
